# From clinical records to community care: using real-world evidence in Ayurveda

**DOI:** 10.3389/fmed.2026.1895750

**Published:** 2026-09-09

**Authors:** Amritha Sindhu D, Bhargavi N, Viraj Bhatia, Veena P K, Shilpa Babu, Sanketh V. Sharma, Poornima Devkumar, Aswini Mohan L, Yashashwini G, Sahana K, Ashwini Mathur, Narendra Pendse, Darshan Shankar, Prasan Shankar

**Affiliations:** 1 The University of Trans Disciplinary Health Sciences and Technology, Bengaluru, India; 2 Institute of Ayurveda and Integrative Medicine (I-AIM) Healthcare Center, Bengaluru, India; 3 Onesto Consulting, Dublin, Ireland

**Keywords:** Ayurveda, clinical outcomes, integrative medicine, real-world data, real-world evidence

## Abstract

**Background:**

Astute observers of health systems are aware that worldwide there is a clinical trend toward integrative and personalized models. Ayurveda, inherently by its theory and practice, a personalized system of healthcare. Evidence from Ayurvedic clinical settings shows its effectiveness in managing chronic diseases; however, a lack of comprehensive multi-centric and retrospective clinical data hampers the credibility of Ayurveda within research circles. Traditional research, particularly Randomized Controlled Trials (RCTs), is deemed unsuitable for evaluating Ayurveda because these trials focus on standardized interventions and do not account for the personalized and multifaceted nature of Ayurvedic practices. This study intends to demonstrate that routine electronic medical record (EMR) data can effectively be transformed into actionable real-world evidence to support Ayurvedic management of chronic conditions.

**Methods:**

Analysis was done using digitized Electronic Medical Records (EMR) Practo Insta, from the Institute of Ayurveda and Integrative Medicine, Bengaluru (and IIHNO Indore). Five clinical areas were assessed: Cancer (*n* = 36), Oral Mucositis (*n* = 89), Psoriasis (*n* = 38), Diabetes Mellitus (*n* = 65), and Infertility (*n* = 65). We used standardized metrics, including EORTC QLQ-C30, WHO Oral Mucositis grades, PASI, DLQI, and HbA1C, to measure outcomes.

**Results:**

Improvements were observed across all clinical areas. In oncology, global QoL increased by 17%, functional scores rose by 10%, and symptomatic burden dropped by 48%. In Oral Mucositis, at the primary 3-week endpoint 57.3% (51/89) of AGR patients achieved complete Grade 0 resolution versus 0% in PBMT. In Psoriasis, 76.3% of patients showed PASI improvement, and 92.1% reported better QoL. Diabetic participants exhibited noticeable reductions in blood glucose levels, with 85% reporting improved QoL. In infertility management, 58% of female participants successfully conceived within an average of 6 months, alongside improvements in secondary fertility and follicular parameters.

**Conclusion:**

Integrating Ayurveda into mainstream healthcare requires a decisive shift toward robust, data-driven evidence. Evaluating personalized, multi-component interventions solely through conventional RCTs is limited, making the systematic use of Real-World Data (RWD) both necessary and appropriate. Standardizing routine EMR data translates nuanced Ayurvedic assessments into quantifiable metrics, bridging the epistemological gap between ancient clinical wisdom and modern scientific rigor to build the long-term evidence base.

## 1 Introduction

Global health systems are currently navigating a profound and unprecedented structural transition, driven by the escalating crisis of Non-Communicable Diseases (NCDs), rapidly aging demographic profiles, and the increasingly unsustainable economic trajectory of modern medical expenditures (1). This strategic shift has revitalized interest in Traditional, Complementary, and Integrative Medicine (TCIM) as a viable framework for sustainable healthcare. By moving beyond the constraints of episodic, symptom-led care, integrative approaches provide a cost-effective, patient-centered framework for addressing modern public health challenges (2). Among different types of traditional treatment practices that offer an efficient response to Non-Communicable Diseases (NCDs), Ayurveda stands out with its profound and thoroughly developed theory. According to the WHO, the United Nations’ Sustainable Development Goal (SDG) 3.4 aims to reduce premature mortality from NCDs by one third by 2030 to advance mental health and wellbeing. Moreover, the integration of traditional medicine into the healthcare system serves as a strategy for achieving Universal Health Coverage (UHC) and meeting SDG 3 objectives (3).

Driven by a growing global interest in holistic health frameworks, the utilization of complementary and alternative medicine (CAM) has accelerated significantly. According to the World Health Organization, these modalities are now utilized by 70–80% of populations within developed nations (4). Recognizing this immense therapeutic and economic potential, the World Health Organization (WHO) has been instrumental in advocating for the integration of traditional medicine into the conventional primary healthcare delivery systems to achieve UHC. Some of these efforts include the creation of the WHO Global Traditional Medicine Center (GTMC) as well as enactment of the WHO Global Traditional Medicine Strategy 2025–2034, whereby global health governance seeks to build an evidence base that will legitimize the practices while at the same time setting up regulatory frameworks for the safe integration of TCIM interventions in National health architecture around the world (2, 5, 6).

Ayurveda brings over several centuries of refined clinical wisdom and extensively documented theoretical frameworks. Beyond its philosophical underpinnings, Ayurveda offers practical approaches to managing chronic illnesses and enhancing resilience against disease (2). Despite the profound wisdom and vast knowledge preserved in classical Ayurvedic literature, the public domain currently lacks systematic data demonstrating its real-world application and reproducible clinical outcomes. Furthermore, practitioners operate without standardized treatment protocols. While some initial progress has been made, comprehensive, open-access documentation regarding pharmacoepidemiology, pharmacovigilance, and adverse drug reactions remains largely unavailable (7).

The fundamental challenge preventing the widespread integration of Ayurveda into mainstream healthcare does not stem from an absence of clinical success, but rather from the rigid methodological hierarchies that currently govern evidence-based medicine. Contemporary biomedical research heavily prioritizes the Randomized Controlled Trial (RCT) as the ultimate gold standard for therapeutic validation (8, 9). Clinical Trials are meticulously designed to maximize internal validity by enforcing strict patient eligibility criteria, controlling for innumerable confounding variables, and utilizing rigid, uniform treatment protocols. This approach is highly effective for isolating the specific, linear efficacy of a single pharmaceutical molecule under highly controlled, idealized clinical conditions (9). By isolating single variables, RCTs inherently exclude the heterogeneous, multifaceted realities of routine clinical practice. They frequently exclude elderly patients, individuals with multiple comorbidities, and those on concurrent medications, thereby limiting the generalizability of the findings to real-world populations (10).

In Ayurveda, this methodological friction is even more profound due to its core philosophical approach of individualization. Ayurvedic diagnostics and therapeutics are inherently personalized, taking into account *Prakriti* (body constitution), *Vikriti* (current pathological state), *Sara* (quality of dhatus), *Samhanana* (compactness of the body), *Pramana* (body or organs measurements), *Satmya* (dietary and environmental compatibility), *Satva* (psychological strength), *Ahara shakti* (digestive capacity), *Vyayama shakti* (exercise tolerance), and *Vaya* (age) (11). Because clinical decision-making relies entirely on this multidimensional assessment to determine a patient’s strength, the specific prognosis of the disease, and the precise suitability of pharmacological interventions, no two patients presenting with the same biomedical diagnosis will receive an identical Ayurvedic prescription (12). Evaluating such complex, multi-component interventions through the rigid lens of conventional RCTs is inadequate (13).

To overcome these structural limitations, researchers are increasingly adopting the paradigm of Whole Systems Research (WSR) (10). WSR provides a robust alternative methodology designed specifically for evaluating complementary and alternative medicine interventions that are too complex for conventional reductionist designs (14). WSR acknowledges the emergent properties of complex adaptive systems, focusing on the relationship between multiple clinical variables and assessing patient outcomes at multiple interconnected levels. The objective-driven focus of WSR aligns seamlessly with Ayurveda, permitting a non-hierarchical approach to evidence generation that authentically represents Ayurvedic practice at the point of care while maintaining scientific rigor (10).

To bridge the efficacy-effectiveness gap and validate the complex realities of traditional medicine within the framework of Whole Systems Research, global health researchers and regulatory bodies are increasingly turning toward Real-World Data (RWD) and its analytical derivative, Real-World Evidence (RWE) (15). RWD encompasses the vast, continuous streams of patient health status and healthcare delivery data routinely collected during everyday clinical practice. This data is extracted from electronic health records (EHRs), patient registries, insurance claims, billing databases, and advanced digital health technologies (16).

When this raw observational data is systematically aggregated, cleaned, and rigorously analyzed, it generates RWE, which provides scientifically valid insights into the actual effectiveness, safety profiles, and long-term clinical outcomes of interventions across diverse, heterogeneous patient populations (16).

FDA has released “Framework for FDA’s Real World Evidence Program” in 2018, document outlines the structured approach to assessing if specific RWD is “fit for use” ensuring data quality, and determining whether a non-interventional or hybrid study design can provide adequate scientific evidence to definitively answer a regulatory question (17). The FDA now actively supports pragmatic clinical trials containing elements that closely resemble routine clinical practice, and hybrid trials that supplement traditional clinical data with EHR and medical claims data (18). Parallel to the FDA’s regulatory evolution, the European Medicines Agency (EMA) and the broader European medicines regulatory network established a massive, unified capability to integrate RWD into continental health policy (19).

The strategic adoption of RWE presents multiple high-order advantages for the scientific validation of Ayurveda. First, RWE captures the exact, unvarnished reality of patient behavior and system-level interactions. It accounts for treatment adherence, concurrent dietary habits, the nuanced adjustments made by physicians over time, and the multi-component nature of prescribed care variables that are nearly impossible to model accurately in an RCT environment (20).

Second, RWE offers a highly pragmatic and cost-effective mechanism for clinical research. Given that bringing a single new biomedical drug to market costs an estimated average of $2.6 billion and requires thousands of patients over several years, leveraging pre-existing, continually generated clinical data from established Ayurvedic hospitals provides an expedited, economically viable pathway to establishing strong baseline evidence for whole-system protocol (21).

Crucially, the global scientific community is increasingly recognizing that RWE should not be viewed as an inferior alternative to RCT data; rather, the two methodologies are mutually complementary. While RCTs remain unmatched for establishing specific, isolated biological mechanisms of action and maximizing internal validity, RWE possesses far superior external validity and generalizability. By standardizing the everyday clinical records gathered from digital health networks, practitioners can effectively translate nuanced traditional qualitative assessments into quantifiable, scientifically interpretable metrics. Converting continuous RWD into robust RWE thus serves as the definitive bridge across the epistemological divide separating ancient clinical wisdom from modern scientific (16).

In India, efforts are already underway to digitize traditional medicine through the Ayush Grid, which serves as a four-tiered digital IT backbone for the entire sector. Its integrated ecosystem includes A-HMIS for clinical data and EHR management, the NAMASTE Portal for standardized ICD-11 terminologies, e-Aushadhi for medicine supply chains, and the Ayush Suraksha Portal for pharmacovigilance (22). Concurrently, practice-based initiatives like the RUDRA Programme (Random Uninterrupted Documentation for Retrospective Analysis) have pioneered the systematic, structured capture of clinical outcomes across multi-institutional Ayurvedic hospital networks (23).

This paper is therefore offered not as a definitive clinical evaluation of any single condition, nor as a formal methodological paper, but as a demonstration of a way of thinking. Across five diverse conditions, we illustrate how the data already generated in everyday Ayurvedic practice can, with modest documentation discipline, be turned into structured real-world evidence. The conditions were chosen to span different kinds of outcomes, from quality-of-life indices, a clinical grading scale, a comparative arm, metabolic markers, to a clinical event, precisely to show the breadth of what routine practice can capture. Our aim is to make this approach feel natural and within reach for practitioners, and to encourage its wider, more systematic adoption.

## 2 Materials and methods

### 2.1 Data source

Data were obtained from two sources: (i) the Practo Insta Electronic Medical Record (EMR) system of the Institute of Ayurveda and Integrative Medicine (IAIM), Bengaluru, and (ii) paper-based clinical case records from IIHNO, Indore for the Oral Mucositis cohort.

### 2.2 Reporting guidelines

This study utilizes routinely collected real-world health data (RWD) and is reported in compliance with the Reporting of studies Conducted using Observational Routinely-collected health Data (RECORD) guidelines (24), which extend the standard STROBE statement.

### 2.3 Data capture and documentation framework

To generate real-world evidence from real-world data, the physicians have used a unified clinical and documentation framework. This study is a retrospective analysis of routinely collected clinical data. All treatment decisions were made as part of routine clinical practice before commencement of the present study. The investigators did not influence treatment allocation, patient management, or clinical decision-making for research purposes.

#### 2.3.1 Treatment options

Depending on the presenting conditions, treatments were either standardized for all patients or personalized for each. The physicians combined traditional Ayurvedic care with standard medical tests (like blood tests) to set a clear baseline for each patient. Individualized treatment decisions were made by the treating physicians based on patients’ clinical presentation, disease severity, patient preferences, and routine Ayurveda clinical judgment. No treatment allocation or clinical management was influenced by the present retrospective analysis.

#### 2.3.2 Real-time data entry

Although clinical management was individualized according to routine clinical practice, the data collection framework remained standardized across all cohorts through the digital documentation platform. Baseline clinical consultations, patient demographics, and initial screening parameters were systematically managed within the Practo Insta EMR platform, whereas the administration of disease-specific diagnostic instruments and longitudinal outcome tracking were executed concurrently utilizing custom-built Google Forms.

#### 2.3.3 Data organizing

Final records were extracted from the Practo Insta EMR system and the Google Forms database. For the Oral Mucositis cohort, paper-based case records from IIHNO, Indore were entered into Microsoft Excel. Datasets from all sources were harmonized using identical variable definitions and outcome measures before being integrated into a single analytical dataset for statistical analysis.

### 2.4 Cohort selection

**FIGURE 1:**
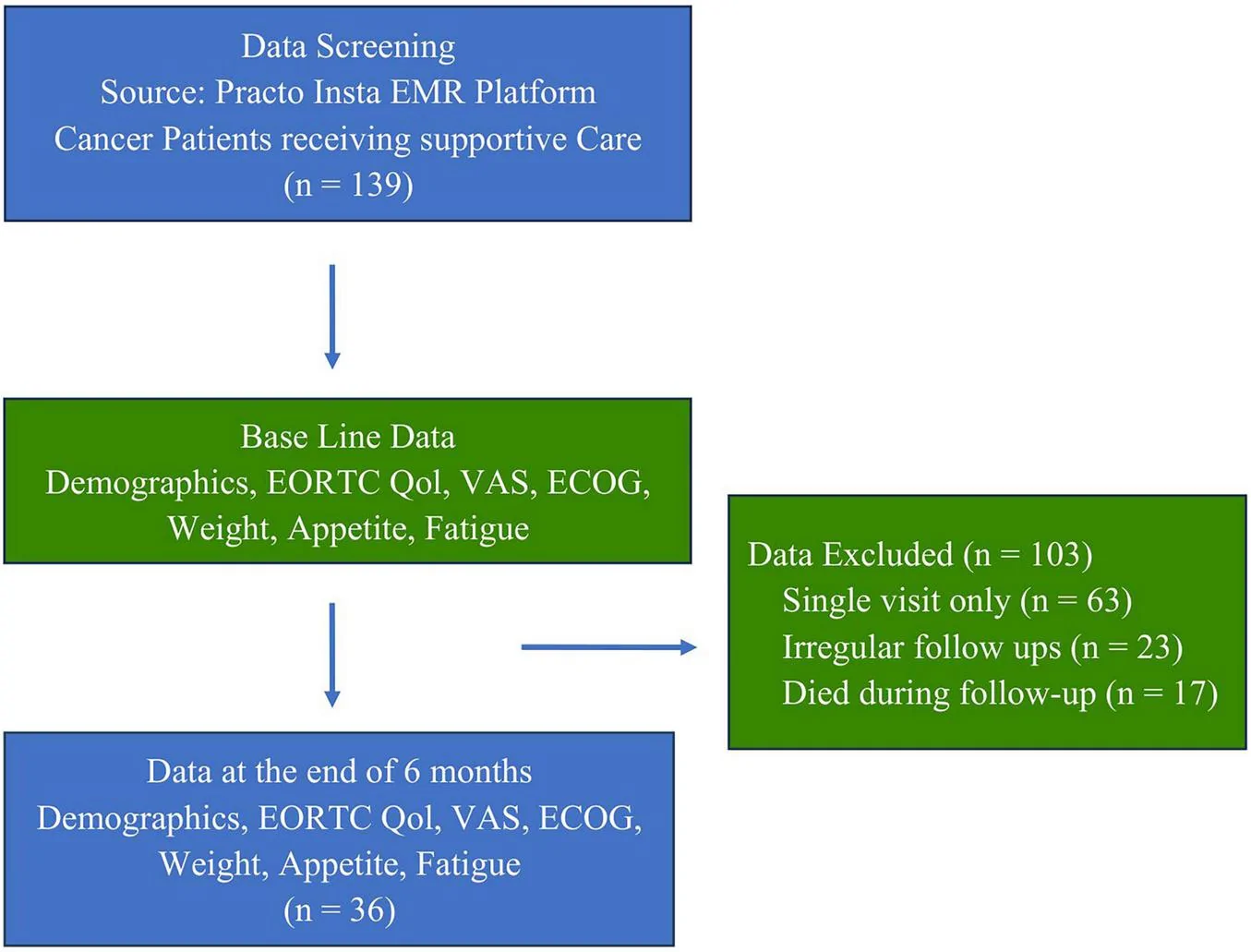
Participant flow and attrition diagram.

**FIGURE 2:**
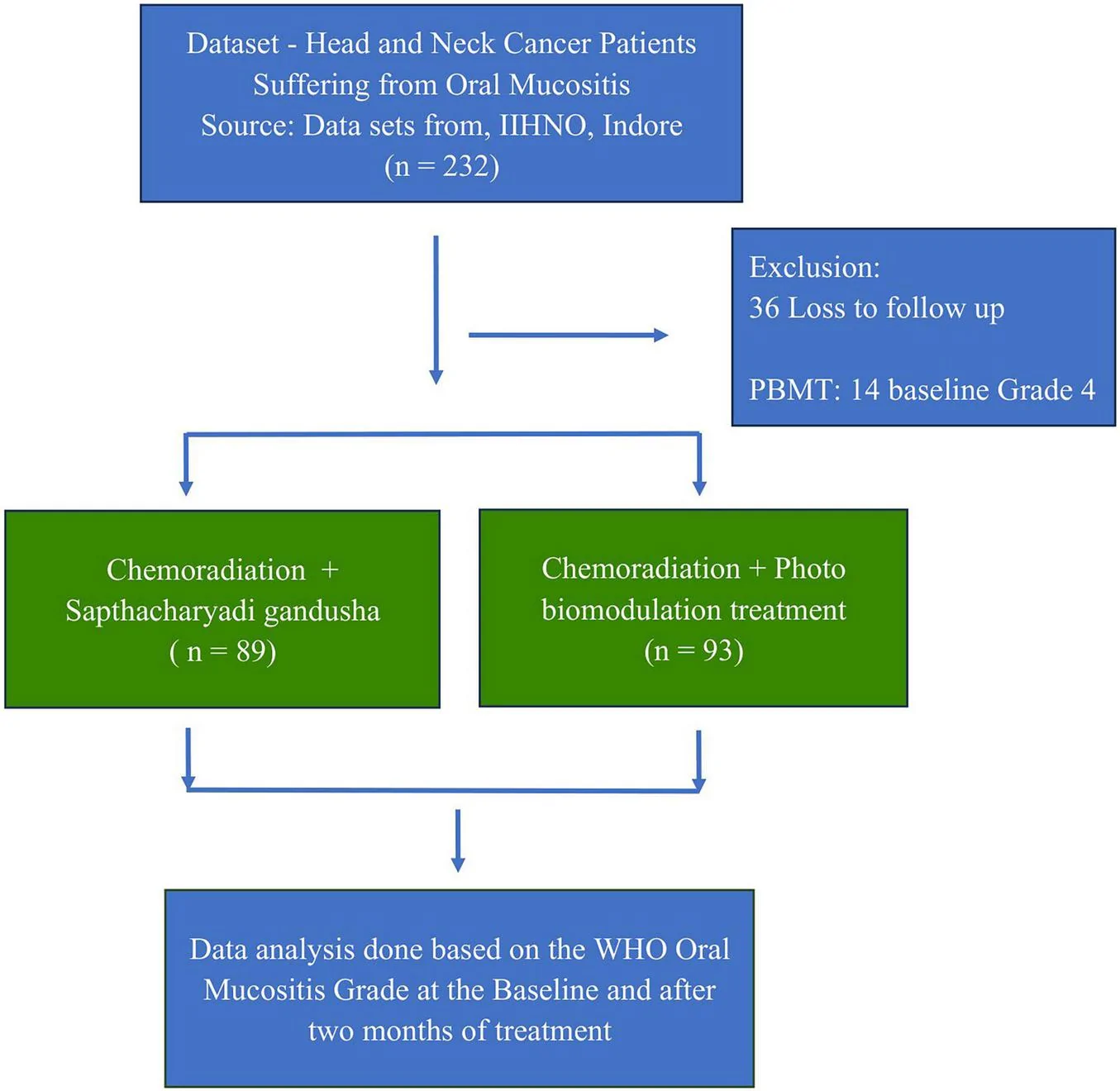
Data extraction and treatment groups for oral mucositis.

**FIGURE 3:**
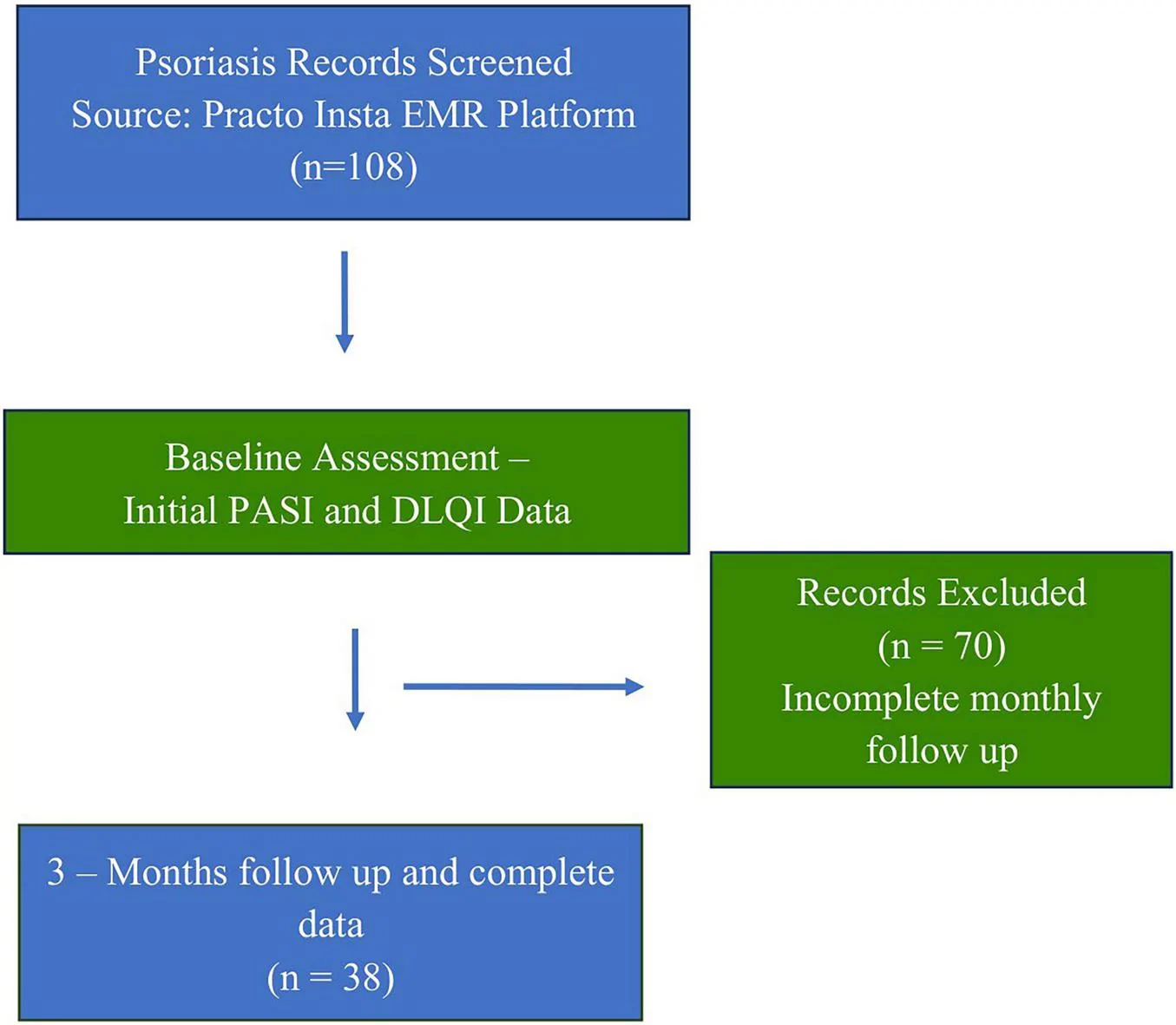
Data extraction and patient evaluation flow for the psoriasis cohort.

**FIGURE 4:**
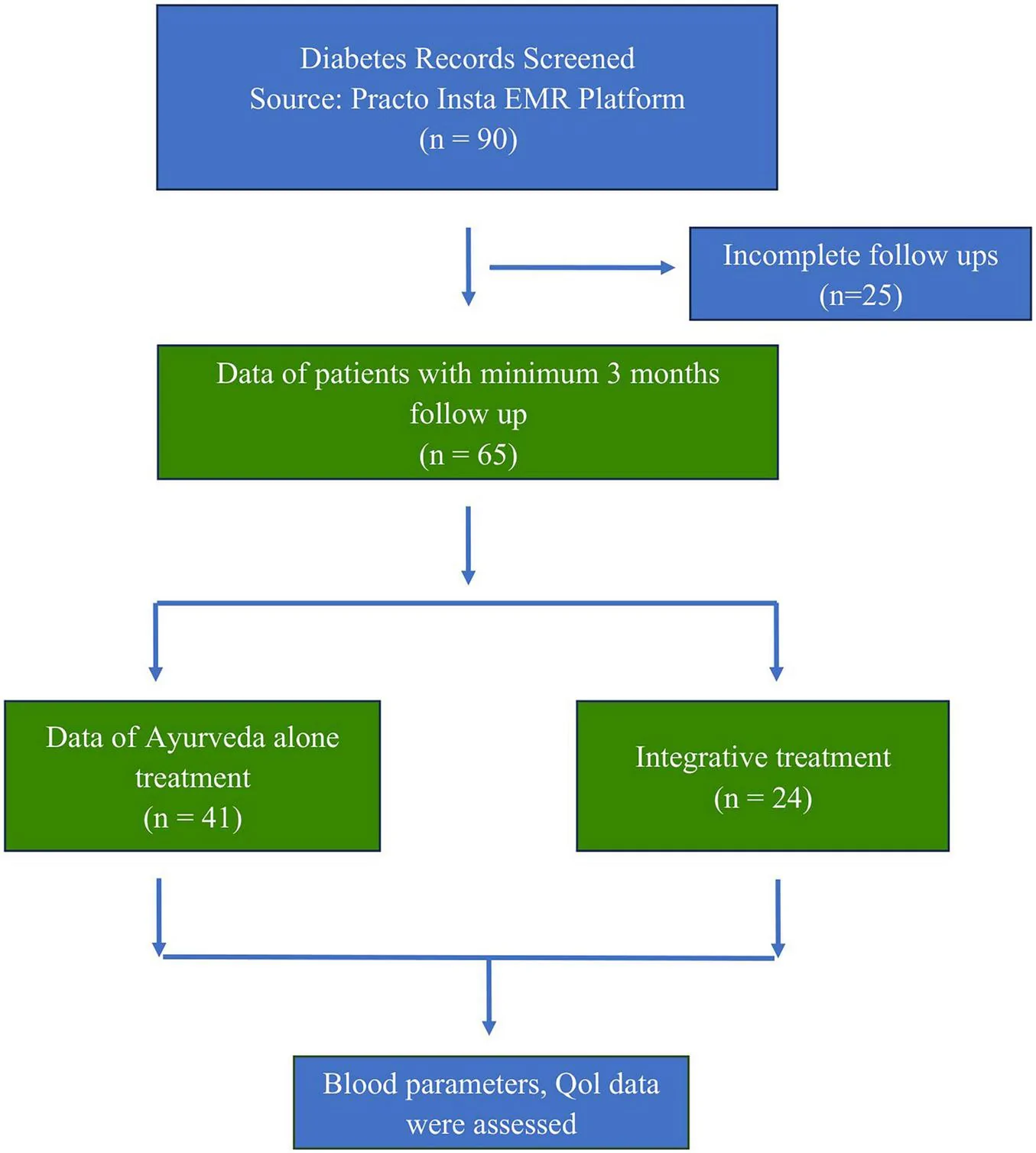
Data extraction and treatment groups for the diabetes patient’s data.

**FIGURE 5:**
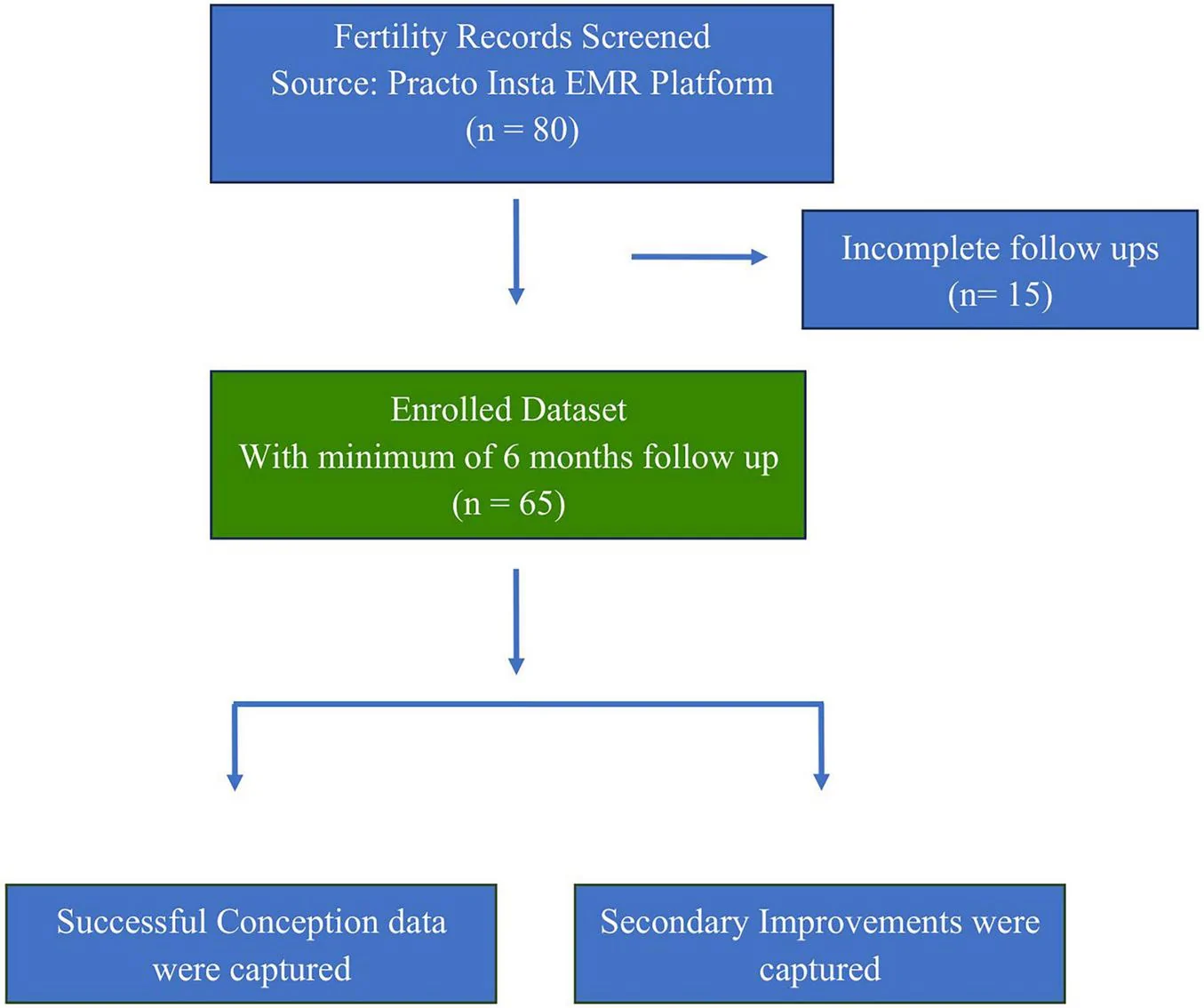
Data extraction and treatment outcomes for the fertility program patient’s data.

Eligible patient records were retrospectively identified from the Practo Insta Electronic Medical Record (EMR) system and digitized clinical records using predefined disease-specific inclusion and exclusion criteria. Records were screened for completeness of baseline characteristics, availability of primary outcome measures, and the predefined minimum follow-up duration. Patient records which did not satisfy the eligibility criteria or had incomplete follow-up data were excluded from the final analysis. The patient record identification, screening, inclusion, exclusion, and final cohort selection process for each clinical condition is illustrated in Figures 1–5.

As this was a retrospective real-world evidence study, no formal sample size calculation was performed. Instead, all eligible patient records meeting the predefined inclusion criteria during the study period were included in the analysis, representing a convenience sample of all available eligible cases.

### 2.5 Records from the five distinct clinical conditions were included

To generate real-world evidence from real-world data, patient health status and clinical progression were systematically captured utilizing validated, condition-specific assessment instruments. They are as follows

#### 2.5.1 Supportive care in oncology

This included records of patients with confirmed cancer who received personalized Ayurvedic supportive care for at least 6 months, detailed list of medicine used in cancer supportive care is mentioned in Supplementary Table 1. Data were captured using Practo Insta, Google Form with EORTC QLQ-C30 questionnaire (25) for measuring quality of life scores, sleep disturbance, fatigue and appetite, Visual Analog Scale (VAS) for measuring the pain, performance status using the ECOG scale, as well as anthropometric measurements, were measured at baseline and every 2 months to track changes in patient quality of life. Details of the data selection are explained in Figure 1. Data were captured between July 2023 and April 2025.

#### 2.5.2 Oral mucositis management

This group included records of head and neck cancer patients with histologically confirmed squamous cell carcinoma undergoing concomitant chemoradiation (IMRT 60–70 Gy). Patients with WHO OM Grade ≥ 1 were given the option to choose between the Ayurveda Gargle Regimen (AGR) or Photobiomodulation Therapy (PBMT). Those who chose AGR received *Sapthachadadi Gandoosham Kashayam* (10 mL, five times daily) plus topical *Panchathikthakam Ghrutham* twice daily; those who chose PBMT received treatment via the Novaduolase 16-Array Cluster device (660 nm and 810 nm; 4–8 J/cm^2^; 3–5 sessions/week). Both were continued for up to 6 weeks or until complete resolution. Patients with baseline WHO Grade 4 OM (PBMT only, *n* = 14) and those lost to follow-up after the initial visit (PBMT only, *n* = 36) were excluded; the final analytic sample was 182 patients (AGR *n* = 89; PBMT *n* = 93). Data were captured between December 2022 and December 2024 (PBMT) and September 2023 and April 2025 (AGR). WHO Oral mucositis grade was assessed at baseline, week 1, and week 3 (primary outcome timepoint), with additional follow-up at 4 months (AGR) and months 1–2 (PBMT), using the WHO oral mucositis grading scale (25). Details of the data selection are explained in Figure 2. All patients in both the Ayurveda Gargle Regimen (AGR) and Photobiomodulation Therapy (PBMT) arms were treated at a single site, IIHNO, Indore, a head and neck oncology center. As IIHNO does not have Ayurveda physicians on staff, the Ayurveda Gargle Regimen was administered under the direct clinical oversight of IAIM Bengaluru’s Ayurveda physicians, who monitored treatment delivery for this arm.

#### 2.5.3 Psoriasis management

This included records of patients with confirmed psoriasis who received personalized Ayurvedic care for at least 3 months, detailed list of medicine used in Psoriasis is mentioned in Supplementary Table 2. PASI (26) and DQLI (27) were assessed at baseline and after three months, details of the data selection are explained in Figure 3. Data were captured between July 2023 and July 2025.

#### 2.5.4 Diabetes mellitus management

Records of patients with a confirmed diagnosis of Diabetes Mellitus managed either exclusively with Ayurveda or through integrative care (Ayurveda combined with conventional medicine), patients received personalized Ayurvedic care with monthly follow-up for an average of 12 months, detailed list of medicine used in Diabetes Management in mentioned in Supplementary Table 3. FBS, PPBS, HbA1C and Quality of life using Quality of Life Instrument for Indian Diabetes Patients (QOLID) were measured at baseline and after 3 to 12 months. Data were captured between July 2023 and December 2025. Details of the data extraction are explained in Figure 4.

#### 2.5.5 Infertility management

Records of patients who had been enrolled in the hospital’s Ayurvedic fertility program and received personalized Ayurvedic care with a minimum follow-up of 6 months were included, detailed list of medicine used in infertility supportive care in mentioned in Supplementary Table 4. Treatment outcomes were recorded. Data were captured between July 2023 and May 2026. Details of the data extraction are explained in Figure 5.

### 2.6 Data access and cleaning methods

Records of participants with a full set of primary outcome data and baseline characteristics were included for final reporting. Furthermore, only records of patients with a pre-defined minimum follow-up duration were retained. Duplicate records were screened before analysis. Data extracted from Practo Insta, Google Forms, and digitized case records were cross-verified for completeness and consistency. Only records with complete primary outcome measurements and the predefined minimum follow-up duration were retained in the final analytical dataset.

In the cancer cohort, patients who died during follow-up or did not complete the predefined assessment schedule were excluded from the final paired outcome analysis due to the unavailability of complete follow-up outcome data.

### 2.7 Ethical permission

Ethical permission was taken from the Institute’s Ethical Committee.

### 2.8 Statistical assessment

#### 2.8.1 Supportive care in oncology

Within-patient changes from baseline to follow-up were tested using the Wilcoxon signed-rank test (non-parametric paired test), as the normality of difference scores could not be assumed in this small sample (*n* = 36). The *p*-values reported in Table 1 correspond to this test. Records with missing outcome data at follow-up were excluded from the analysis (complete-case analysis); the number of patients contributing to each comparison is *n* = 36 unless otherwise stated. Given the exploratory and descriptive nature of this real-world evidence study, no adjustment for multiple comparisons was applied across the nine outcome measures in Table 1; all *p*-values should therefore be interpreted as hypothesis-generating rather than confirmatory, and findings require validation in prospectively designed studies.

**TABLE 1:** Outcome measures before and after ayurveda-based supportive care (*n* = 36).

	Baseline (Mean ± SD)	On follow-up (Mean ± SD)	Percentage Change (%)	*p*-value**
EORTC - QLQ - C30				
Functional	70 ± 22.13	77.4 ± 18.1	+10.5	0.002
Symptomatic	26.62 ± 20.06	13.83 ± 12.18	- 48	< 0.001
Global Qol	61.34 ± 24.61	71.76 ± 16.88	+17	0.002
Sleep Disturbance*	3 ± 0.8	1.8 ± 0.8	- 40	0.005
Improved Appetite*	1.94 ± 0.99	3.37 ± 0.58	+ 42.4	< 0.001
Fatigue*	3 ± 0.80	1.90 ± 0.75	- 36.6	< 0.001
**Pain (VAS)**	5.8 ± 2.59	3.19 ± 2.15	- 45	< 0.001
**Weight**	68.94 ± 14.66	68.14 ± 14.08	- 1.1	N.S***
**ECOG**	1.47 ± 0.86	1.39 ± 0.92	- 5.4	N.S***

*Sleep, appetite, and fatigue rows use a 0–4 clinical rating scale, not the 0–100 EORTC QLQ-C30 subscale, **Wilcoxon signed-rank test, ***N.S, not significant.

#### 2.8.2 Oral mucositis management

Fisher’s exact test assessed the independence of treatment type (AGR vs. PBMT) and WHO OM grade at the primary timepoint of week 3; a chi-square test assessed baseline comparability. Propensity scores were estimated using logistic regression with age, sex, and baseline OM grade as covariates, with four within-class comparisons performed. The final analytic sample was 182 patients (AGR *n* = 89; PBMT *n* = 93), all with complete WHO OM grade data at week 3. Patients excluded prior to analysis (14 baseline Grade 4 PBMT; 36 lost to follow-up PBMT) are represented in the patient flow diagram (Figure 2); no imputation was required. No adjustment for multiple comparisons was applied, as a single primary inferential comparison was pre-specified for this cohort.

#### 2.8.3 Psoriasis management

Within-patient changes from baseline to 3 months were tested using the Wilcoxon signed-rank test. Only records with complete PASI and DLQI data at both time-points were included (complete-case analysis; *n* = 38). No adjustment for multiple comparisons was applied across the two co-primary outcome measures (PASI and DLQI); results are exploratory.

#### 2.8.4 Diabetes mellitus management

Within-patient changes from baseline to the end of the observation period (minimum 3 months; average 12 months) were tested using the Wilcoxon signed-rank test for each glycaemic parameter. Only records with paired baseline and follow-up values for each parameter were included in the respective comparison (complete-case analysis). Given that three metabolic endpoints (FBS, PPBS, HbA1C) were tested simultaneously, a Bonferroni-corrected significance threshold of *p* < 0.017 is recommended when interpreting these results; all reported *p*-values remain exploratory pending prospective confirmation.

#### 2.8.5 Infertility management

No inferential statistical test was applied for this cohort, as there was no comparator group; analysis is purely descriptive. Patients who did not complete the minimum 6-month follow-up (*n* = 15) were excluded prior to analysis (complete-case analysis; *n* = 65). No adjustment for multiple comparisons was required given the descriptive nature of the analysis.

All statistical analyses were performed using SPSS. Statistical significance was considered at *p* < 0.05 unless otherwise specified.

## 3 Results

To present real-world evidence from our hospital data, we are presenting the results for five different health conditions separately. Looking at each condition on its own provides a clear, practical view of how real patients respond to their treatments in an everyday hospital setting.

### 3.1 Cancer care

This cohort illustrates how a validated multi-domain quality-of-life instrument (EORTC QLQ-C30) can be captured longitudinally in routine practice. The study initially enrolled 139 patients, with 36 (excluded 103 patients: 63 patients had only single visits, 23 patients were irregular for follow ups, and 17 patients had passed away) patients completing follow-up to be included in the final analysis; others were excluded due to death, non-adherence, or being lost to follow-up. The participants’ mean age was 61.8 years (SD = 13.4), 69% were females, and 31% were males. The majority of patients were treated for breast cancer, followed sequentially by colon, cervical, and prostate cancers. Pre- and post-treatment outcome measures are represented in Table 1. Concurrent treatment status at enrolment was mixed: 6 patients (17%) were undergoing active chemotherapy/radiotherapy, 18 (50%) had recently completed chemotherapy/radiotherapy, 9 (25%) were receiving palliative care, and 3 (8%) were receiving Ayurveda as primary treatment with no concurrent conventional cancer therapy.

### 3.2 Oral mucositis

This cohort illustrates two things at once, a standardized clinical grading scale (WHO OM) captured in routine oncology practice, and a comparator arm structured from patient-choice EMR data. All patients received concurrent chemoradiation. After exclusions (PBMT: 14 baseline Grade 4; 36 lost to follow-up), 89 patients received the Ayurveda Gargle Regimen (AGR) comprising *Sapthachadadi Gandoosham Kashayam* gargle plus topical *Panchathikthakam Ghrutham*, and 93 patients received PBMT. AGR patients ranged in age from 24 to 84 years (median 50; 72 males, 17 females); the majority were aged 40–50 (32/89). PBMT patients ranged from 27 to 87 years (median 52; 69 males, 24 females); the majority were aged 50–60 (27/93). Primary outcome WHO OM grade results at week 3 for both groups are detailed in Table 2.

**TABLE 2:** WHO oral mucositis grade distribution at week 3: AGR (*n* = 89) vs. PBMT (*n* = 93).

Group	Status	*n*	%	OM grade	*n*
AGR (*n* = 89)	OM improved	80	89.9	Grade 0	51
				Grade 1	27
				Grade 2	2
	OM not improved	9	10.1	Grade 1	3
				Grade 2	6
PBMT (*n* = 93)	OM improved	14	15.05	Grade 1	8
				Grade 2	6
	OM not improved	75	80.65	Grade 1	11
				Grade 2	30
				Grade 3	34
	OM worsened	4	4.3	Grade 2	2
				Grade 3	2

By the end of the second month, 89.89% (80/89; 95% CI: 81.67–95.27%) of patients in the Ayurveda group showed clinical improvement, with all 80 achieving Grade 0 status, indicating complete resolution of oral mucositis. In contrast, within the PBMT group, 15 % (14/93; 95% CI: 9.3–23.3%) demonstrated clinical improvement.

### 3.3 Psoriasis management

This cohort illustrates how a disease-specific severity index (PASI) can be paired with a patient-reported QoL measure (DLQI) to give a dual perspective on treatment response. Here, of 108 patients’ records screened, 38 patients underwent treatment for an average of 3 months with regular monthly follow-ups; Among those 38, 20 patients were males, and 18 were females. The majority of them belonged to the 30 to 40-year age group.

All the patients completed the assessment using the PASI and DLQI Questionnaire. A marked decrease in PASI and DLQI scores was observed over three months, consistent with an association between the Ayurvedic intervention and improvements in psoriasis severity and quality of life.

**FIGURE 6:**
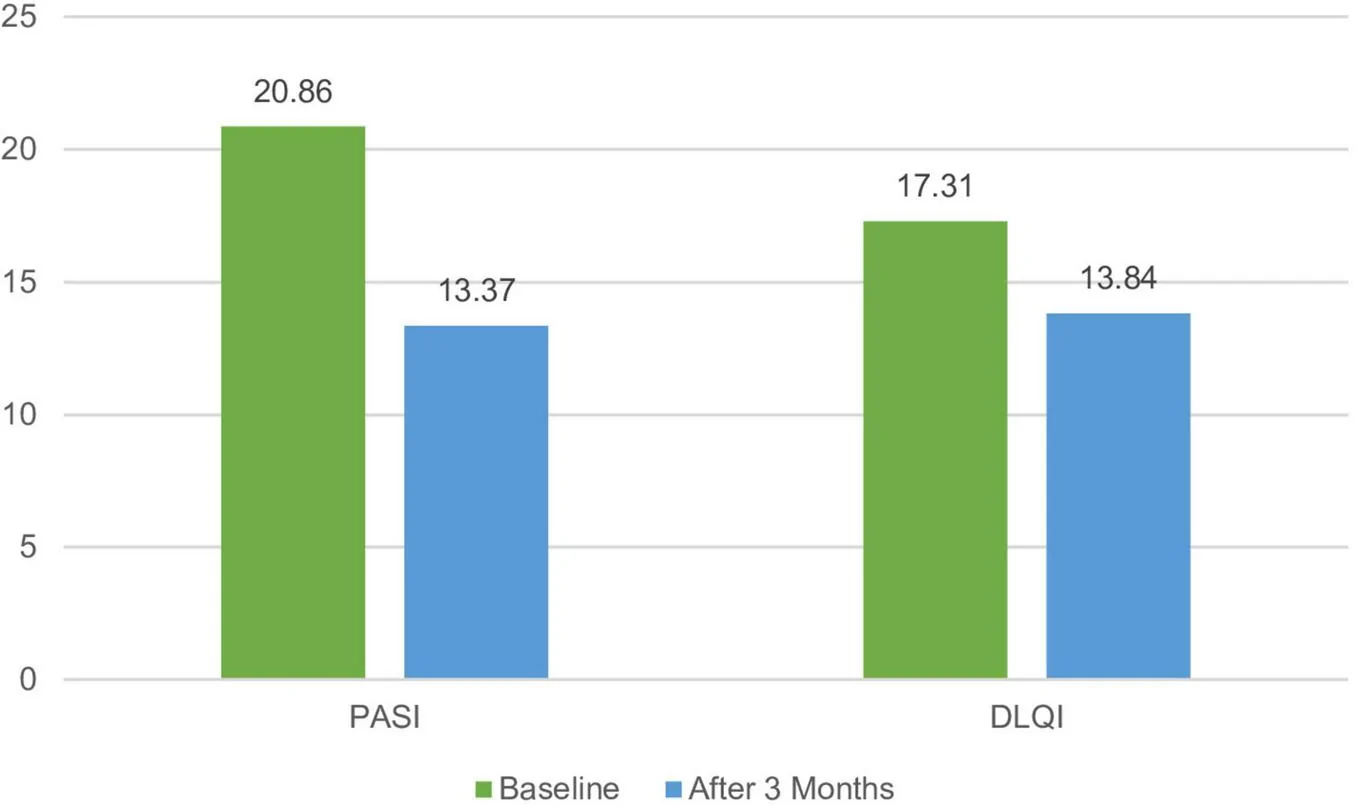
Mean PASI and DLQI status at baseline and 3 months follow-up of psoriasis patients (*n* = 38). PASI, Psoriasis Area Severity Index; DLQI, Dermatology Life Quality Index.

Results showed that 76.32% (29/38; 95% CI: 59.76 to 88.56%) of patients demonstrated PASI improvement, with mean PASI decreasing from 20.86 ± 2.76 to 13.37 ± 1.88 post-treatment. These findings are presented in Figure 6. Furthermore, 92.11% (35/38; 95% CI: 78.62 to 98.34%) of patients reported an improved Quality of Life (QoL), with the mean DLQI score reducing from 17.31 ± 1.13 at baseline to 13.84 ± 1.31.

### 3.4 Diabetes mellitus

This cohort illustrates how laboratory-based metabolic markers (FBS, PPBS, HbA1c) can be tracked from routine EMR records over a multi-month treatment course. A total of 90 patient records were screened for diabetes, and 65 patients were enrolled. The study population included 43 males and 22 females, with ages ranging from 24 to 71 years (mostly between 40 and 59 years old). Looking at the treatment frameworks, 63.08% (41/65; 95% CI: 50.20 to 74.72%) received Ayurveda alone, while 36.92% (24/65; 95% CI: 25.28 to 49.80%) received integrative care with conventional medicine.

**FIGURE 7:**
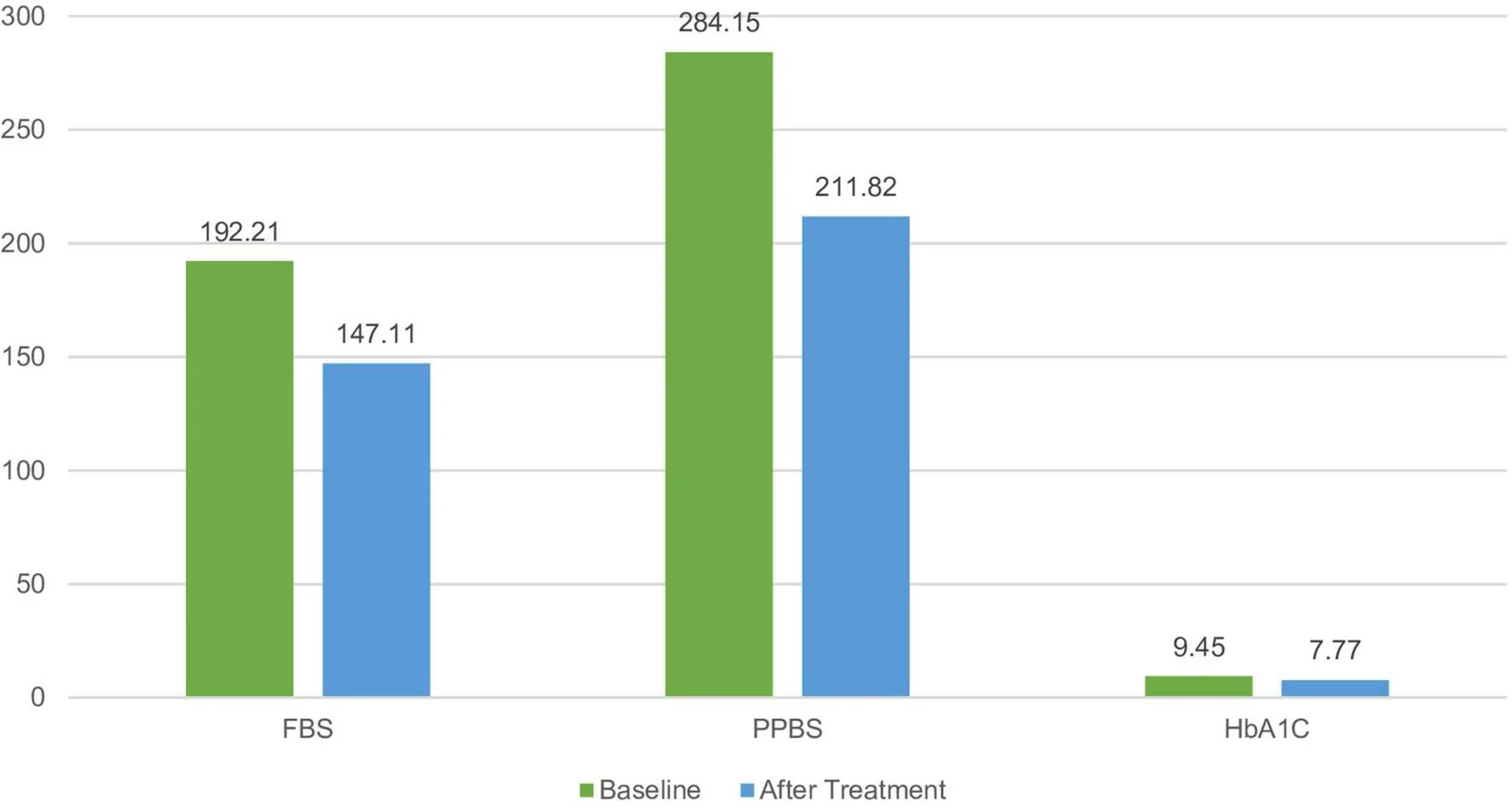
Before and after mean values of blood parameters in diabetic patients (*n* = 65). FBS, fasting blood sugar; PPBS, post-prandial blood sugar.

The average treatment duration was 12 months with regular monthly follow-ups, showing a reduction in diabetic blood parameters. These findings are presented in Figure 7. The mean Quality of Life (QOLID) score improved from 85.67 ± 12.37 before treatment to 94.30 ± 7.96 after treatment. Quality of life (QOLID) improved in 84.62% (55/65; 95% CI: 73.52 to 92.37%) of patients, maintained in 10.77% (7/65; 95% CI: 4.44 to 20.94%), and decreased in 4.62% (3/65; 95% CI: 0.96 to 12.90%).

### 3.5 Infertility management

This cohort illustrates how a discrete clinical event (conception) can be captured as a real-world outcome from routine fertility-program records. A total of 80 participants were initially screened for the Ayurvedic fertility program, and 65 female patients were enrolled.

**FIGURE 8:**
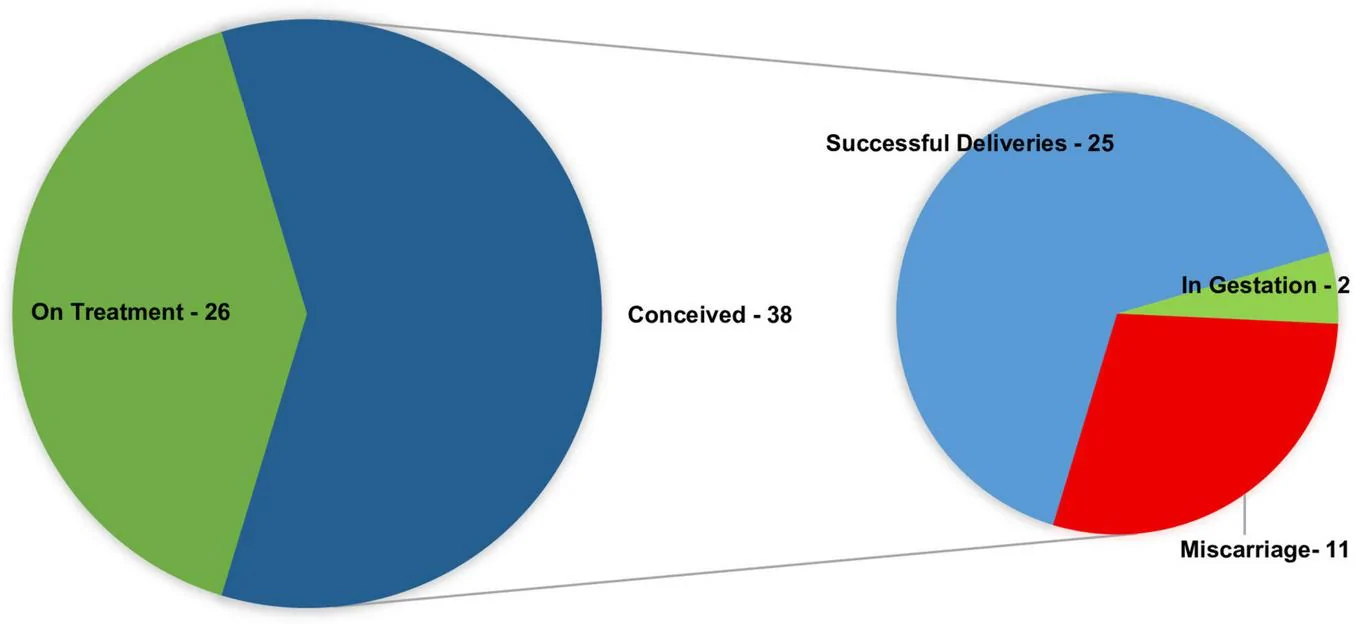
Conception and pregnancy outcomes (*n* = 65).

These patients, evaluated as of May 2026, ranged in age from 23 to 46 years. Following an average treatment period of six months, 38 out of 65 patients (58.46%; 95% CI: 45.56–70.56%) achieved successful conception. These findings are presented in Figure 8. Of these, 11 (28.95%; 95% CI: 15.42%–45.90%) had concurrent IVF treatment. In addition, 26 out of 65 participants (40.00%; 95% CI: 28.04%–52.90%) showed significant improvement in secondary fertility parameters. These clinical enhancements included the correction of anovulation, the regularization of menstrual cycles, increased endometrial thickness, improved follicular parameters, restoration of tubal patency, and the optimization of essential hormonal profiles (FSH, LH, AMH, progesterone, and prolactin).

## 4 Discussion

### 4.1 Clinical outcomes and therapeutic potential

The present analysis illustrates, across five conditions, how routinely-collected Ayurvedic EMR data can be structured into interpretable clinical outcomes. Findings from our dataset demonstrated clinically meaningful improvements across Cancer, Oral Mucositis, Diabetes, Psoriasis, Infertility, reinforcing Ayurveda’s potential as a holistic and individualized healthcare approach.

In the cancer care, improvements were observed in overall quality of life (QOL) and symptom management, reflected by enhanced functional capacity and reductions in pain, fatigue, and sleep disturbances. However, the oncology cohort experienced substantial attrition due to patient mortality and incomplete follow-up, which may have introduced attrition bias and limited the generalizability of these findings. These findings therefore require confirmation through larger prospective studies. In oral mucositis management, Patients receiving AGR showed more favorable clinical outcomes than those receiving PBMT at the primary week-3 endpoint: 57.3% of AGR patients achieved complete Grade 0 resolution compared with 0% in PBMT (*p* < 0.0001); at 4-month follow-up this rose to 89.9% for AGR. These observations suggest a possible association between integrative Ayurvedic supportive care and improved symptom management, warranting further prospective investigation.

Similarly, improvements observed in metabolic health strengthen the growing evidence for Ayurveda’s role in chronic disease management. Reductions in blood glucose parameters were observed following individualized Ayurvedic management. In dermatological care, psoriasis patients experienced notable reductions in disease severity alongside improvements in quality of life following a 3-month treatment period, emphasizing the relevance of patient-centered and long-term approaches in chronic inflammatory conditions. Reproductive health outcomes were equally encouraging, with 38 out of 65 infertile patients successfully conceived and 25 patients have delivered healthy babies, suggesting a possible association between individualized Ayurvedic care and improved reproductive outcomes. These observations resonate with the broader literature on Whole Systems Research (WSR), which advocates studying Ayurveda as an integrated, multi-component intervention rather than isolating individual therapeutic components.

Existing literature further supports these findings. Studies among breast cancer survivors receiving whole-system multimodal Ayurvedic interventions have demonstrated reductions in cancer-related fatigue and improvements in overall QOL, while also reporting high feasibility and patient acceptability (28). Similarly, evaluations of the *sapthachhadadi gandoosham* regimen among head and neck squamous cell carcinoma patients reported delayed onset and reduced severity of oral mucositis without adversely affecting locoregional tumor recurrence (29), findings that closely align with our observations.

In dermatology, objective measures such as the Psoriasis Area Severity Index (PASI) have consistently documented meaningful clinical improvement following Ayurvedic therapies. Case-based evidence has reported substantial reductions in PASI scores after comprehensive *Shodhana* and *Shamana* interventions, alongside marked improvements in quality of life and symptom relief (30, 31). Likewise, retrospective evaluations among patients with Type 2 Diabetes undergoing integrated Ayurvedic management, including *Panchakarma*, dietary regulation, and herbal medicines, have demonstrated significant reductions in glycemic markers such as HbA1c and blood glucose levels (32), supporting our metabolic findings. The data on Diabetes shows people are volunteering concurrent treatment, reflecting the unmet need of society to seek alternatives other than Allopathy. This underlines a largely unaddressed policy making gap to facilitate, nourish and guide health-care seeking behavior based on RWE generated from RWD, to shape decision making.

Reproductive health evidence also reflects similar therapeutic promise. A systematic review involving 14 studies (*n* = 248) reported favorable outcomes of Ayurvedic interventions in conditions such as severe PCOD, endometriosis, and oligoasthenozoospermia, indicating substantial restorative potential (33). However, the review also emphasized important methodological limitations, including small sample sizes and heterogeneity in treatment protocols. These limitations highlight the urgent need for rigorous clinical documentation, standardized reporting frameworks, and well-designed prospective studies.

Taken together, the findings from our analysis and supporting literature suggest that digitized clinical repositories can generate meaningful real-world evidence for Ayurveda and help bridge the gap between traditional knowledge systems and contemporary evidence generation. While these outcomes should be interpreted cautiously due to the observational nature of many datasets, they provide a strong rationale for conducting more rigorous, adequately powered clinical investigations to validate effectiveness, explore mechanisms, and strengthen the evidence base for integrative healthcare models.

### 4.2 Methodological shifts: RWD vs. RCTs

The transition towards evidence-based medicine has traditionally prioritized Randomized Controlled Trials (RCTs) as the gold standard. However, the rigid constraints, highly controlled environments, and single-agent focus of RCTs often fail to capture the complexities of actual clinical practice. WSR addresses this by capturing the emergent properties of complex adaptive systems. As methodological pioneers note, evaluating Ayurvedic whole systems often places trials in the middle of the efficacy-effectiveness continuum (such as on the PRECIS scale), serving as a necessary and scientifically valid compromise between strict experimental rigor and the pragmatic realities of individualized clinical care (34).

### 4.3 Bridging the evidence gap

Integrating Ayurveda into mainstream global healthcare necessitates a shift from experience-based practice to data-driven validation. While modern healthcare prioritizes standardized protocols, Ayurveda has historically relied on classical texts and practitioner experience. Real-World Evidence (RWE) bridges this gap by reflecting everyday clinical settings and patient adherence patterns.

It is important to acknowledge that our current results showed varying levels of improvement among patients. Because we utilized a patient-centered approach, confounding factors inherent in general practice were not isolated. Furthermore, while results from our hospital setting are encouraging, data from a single institution is insufficient to claim universal effectiveness. This paper serves as a foundation for generating future RWE across diverse clinical settings to establish broader scientific credibility.

### 4.4 Policy integration and future frontiers

Moving from evidence generation to routine practice, RWD provides the transparency required to seamlessly integrate Ayurveda into mainstream healthcare. By establishing validated and integrative treatment options, health systems can transform traditional medicine into accessible, culturally grounded solutions (34).

India’s National Ayush Mission (NAM) exemplifies this translation by co-locating Ayush services within Primary Health Centers (PHCs). Ultimately, coupling ancient Ayurvedic wisdom with the analytical power of RWE ensures that traditional practices are safely and sustainably integrated into the future of global healthcare. India is currently laying the groundwork for a scalable medical model that balances heritage with modern rigor through AI integration, digital health systems (ICD-TM2), and advanced “Ayurveda Biology” research such as metabolomics (35).

## 5 Conclusion

Ultimately, the value of this work lies not only in the individual clinical findings but also in demonstrating that routinely collected Ayurvedic clinical records can be systematically transformed into Real-World Evidence (RWE). These examples illustrate the feasibility of adopting a structured documentation framework to generate evidence from routine clinical practice. If this encourages more practitioners and vaidyas to document their clinical practice in a standardized and shareable manner, the collective body of real-world evidence can continue to expand, providing a stronger foundation for future multicentric research and the evidence-informed integration of Ayurveda into mainstream healthcare.

## 6 Future directions

To further advance the integration of Real-World Evidence (RWE) in this field, future research should prioritize expanding data collection to a multi-center network of diverse Ayurvedic institutions, thereby enhancing the generalizability of findings and establishing robust benchmarks across varied demographic populations. Longitudinal efforts are also essential; extending follow-up periods beyond 12 months will be critical to evaluating the long-term sustainability (34) and safety profiles of integrated Ayurvedic protocols over several years. Additionally, future studies may focus on the standardization of outcomes through the inclusion of validated patient-reported outcome measures (PROMs) that specifically capture the subjective improvements in quality of life inherent to the Ayurvedic paradigm. Finally, “bridging” studies are needed to compare outcomes across different clinical sites, helping to define a minimum baseline clinical effect and refine standardized interventions for broader clinical application. Future prospective studies should also incorporate randomized or comparator-controlled designs, where feasible, to more clearly distinguish treatment-related effects from natural disease progression or regression to the mean.

## 7 Limitations

The retrospective study design lacked prospective randomization and comparator groups for most clinical cohorts, limiting causal inference regarding treatment effects. Selection bias may be present, as only eligible patient records with complete follow-up data were included. Each disease cohort was single-center in design: the Cancer, Psoriasis, Diabetes Mellitus, and Infertility cohorts were each drawn from a single institutional setting, IAIM Bengaluru, while the Oral Mucositis cohort was drawn from a single site in Indore (IIHNO), under the direct clinical oversight of IAIM Bengaluru’s Ayurveda physicians. This reliance on single-center data for each cohort restricts the generalizability of findings to other Ayurvedic practice environments. High attrition, particularly within the oncology cohort, may have introduced survivorship bias and influenced outcome estimates. Furthermore, individualized Ayurvedic treatment protocols varied according to patients’ clinical needs, introducing treatment heterogeneity that limits direct comparison of therapeutic effects across participants. As a real-world study, confounders such as dietary adherence, concurrent medications, and lifestyle could not be completely controlled. All complete patient records available were considered for analysis, and no statistical tests were applied to adjust for these external confounding effects.

## Data Availability

The raw data supporting the conclusions of this article will be made available by the authors upon request.
