# Correction: From clinical records to community care: using real-world evidence in Ayurveda

**DOI:** 10.3389/fmed.2026.2009070

**Published:** 2026-10-06

**Authors:** Amritha Sindhu D, Bhargavi N, Viraj Bhatia, Veena P K, Shilpa Babu, Sanketh V. Sharma, Poornima Devkumar, Aswini Mohan L, Yashashwini G, Sahana K, Ashwini Mathur, Narendra Pendse, Darshan Shankar, Prasan Shankar

**Affiliations:** 1 The University of Trans Disciplinary Health Sciences and Technology, Bengaluru, India; 2 Institute of Ayurveda and Integrative Medicine (I-AIM) Healthcare Center, Bengaluru, India; 3 Onesto Consulting, Dublin, Ireland

**Keywords:** Ayurveda, clinical outcomes, integrative medicine, real-world data, real-world evidence

In the acknowledgements, the contribution of Dr. Madhuri Kulkarni, Assistant Professor, Department of Statistics, Savitribai Phule Pune University, who provided statistical guidance for the study, was inadvertently omitted.

The acknowledgements statement should be as follows:

“We would like to thank all participants for their valuable involvement in this study. We also sincerely acknowledge the IIHNO, Indore team for their continuous support, and the Rural India Supporting Trust (RIST) for providing the financial support that made this work possible. We would also like to express our sincere gratitude to Dr. Madhuri Kulkarni, Assistant Professor, Department of Statistics, Savitribai Phule Pune University, for her valuable contribution to the statistical aspects of this study.”

The original version of this article has been updated.

