# Supplementary material for: From clinical records to community care: using real-world evidence in Ayurveda
Source: Front Med (Lausanne). 2026 Sep 9;13:1895750. doi: 10.3389/fmed.2026.1895750 (PMC13613639; doi:10.3389/fmed.2026.1895750)
Supplement: Supplementary file 1 [file Table_1.docx]

**Supplementary Table 1: List of medicines used in Cancer Supportive Care**

| **Internal Medicines** | **External Oils & Ointments** | **Gargle & Oral Rinse** | **Drinking Decoctions & Teas** |
| --- | --- | --- | --- |
| Ashwagandha Churna | Dhanwantharam Tailam | Triphala Kwatha Churna (gargle decoction) | Lakshmana Leaves Tea |
| Shatavari Churna | Ksheerabala Tailam | Spatikam / Alum | Dasamoola Drinking Decoction |
| Guduchi Churna | Balaswagandhadi Oil | Arimedadi Tailam (oil pulling) | Gokshura + Punarnava Decoction |
| Vidarikanda Churna | Sahacharadi Tailam | Oro-T Oral Rinse | Sariba Decoction |
| Abhraka Bhasma | Murivenna | Sapthachadadi Kashayam | Parijatha Tea |
| Chausashti Pippali Powder | Karpooradi Tailam | Mahathiktaka Gritha (kavala) | Sour Sop Leaf Tea |
| Panchakola Churnam | Kottamchukkadi Tailam | Panchavalkala Ks Churna (wound wash) | Vinca Rosea Tea |
| Rasasindooram Cap | Mahamasha Tailam | Salt + Turmeric Gargle | Turmeric + Aloe + Ginger + Amla Concentrate |
| Cruel Plus Cap | Supthi Tailam | Sapthachadadi Ks | Amla + Ginger + Turmeric Sherbet |
| Indukantha Gritha / Cap | Pinda Tailam |  | Tulsi + Ginger + Honey + Dod Patre |
| Makardhwaja Gutika Gold | Chinchadi Oil |  |  |
| Nityananda Ras Tab | Eladi Oil |  |  |
| Kanchanara Gulgulu Tab | Jathyadi Oil |  |  |
| Chandraprabha DS Tab | Dasanga Churna |  |  |
| Amruthadi Gulgulu Tab | Kolakulathadi Churnam |  |  |
| Sallaki XT / MR Tab | Panchavalkala Lepam |  |  |
| Ashwagandharishta | Shatadautham Gritha Ointment |  |  |
| Gokshuradi Guggul | Trichoderm + Cutis Cream |  |  |
| Punarnavadi Ks Tab | Cutis Dusting Powder |  |  |
| Gokshur Kadha / Gokshura Churna | Eladi Cream |  |  |
| Makardhwaja Rasayan Tab | Nimbadi Churnam + Aragwadha (bath wash) |  |  |
| Mahathiktaka Gritha | Sathadautham Gritha Ointment |  |  |
| Vilwadi Lehyam |  |  |  |
| Vasthyamayanthaka Gritha |  |  |  |
| Dadimadi Gritha |  |  |  |
| Panchagavya Gritha |  |  |  |
| Brahma Rasayanam |  |  |  |
| Sanjeevani Vati |  |  |  |
| Samshamani Vati |  |  |  |
| Dhanwantharam Gulika |  |  |  |

**Supplementary Table 2: List of medicines used in Psoriasis**

| **Internal Medicine** | **Local Application** |
| --- | --- |
| Kaisoragulgulu | Asoria Oil |
| Atrisor Cap | Atrisor Shampoo |
| Patolakaturohinyadi Kashayam() | Vaidyaratnam Soap |
| Thikthakam Ghritham | Vettupala tailam |
| G P 500 Cap | D-Sora Lotion |
| Mahathiktaka Gritha | Psorakot Ointment |
| Gandhaka Rasayana | Madhuyashtyadi Thailam |
| D-Sora Tab | D-Sora Soap |
| Manibhadra Guda | Atrisor Moisturizer |
| Aragwadhamahathiktham Ghrutham | Scurfol Lotion |
| Thickshoot Care Cap | Dinesavallaydi Oil |
| Aragwadhamrithadi KS | Moish Lotion |
| Patolamooladi Ks | Jathyadi Gritham Ointment |
| Panchatikta Guggulu Ghrita | Trichoderm Cream |
| Biovestin Tab | Atrisor Ointment |
| Anuloma DS Tab | Jathyadi Gritham |
| Vilwadi Gulika | Kshara Thailam |
| Mahathikthaka Gritham Cap | Mahathikthakam Lepam |
| Gulguluthikthakam Kashayam | Khajitha Pinda Thailam |
| Sonithamrutham Ks | Jeevantiadi Yamakam |
| Avipathi Choornam | Kusuma Bath Powder |
| Mahamanjishtadi Ks |  |
| Aragwadhadi kashayam |  |
| Thikthakam Ks |  |
| Rasasindooram Cap |  |
| Mahathiktaka Ghrita |  |
| Mahathikthaka Gritha |  |
| Patoladi Gritham |  |

**Supplementary Table 3: List of medicines used in Diabetes Mellitus**

| Meha abhyaya kashaya | Dia P |
| --- | --- |
| Nishakathakadi kashayam | Vasantha kusumakara rasa |
| Guggulu tiktaka Kashaya | Chandra prabha vati |
| Manjishtadi kashaya | Mehanil tab |
| Varanadi kashayam | Arogyavardini rasa |
| Ayaskriti | Liv 52 DS |
| Nisa amalaki | Tribang shila |
| Mahakalyanaka gritha | Sumenta tab |
| Dhanwantara taila | Manasamitra vati |
| Pinda taila | Asanadigana paneeya |
| KB taila | Dashamoola + Punarnava paneeya |
| Supti taila | Dhanwantara gritha |
| Triphala + Musta + Haridra churna |  |

**Supplementary Table 4: List of medicines used in Infertility treatment**

| Phalasarpis ghrita | Sukumara ghrita |
| --- | --- |
| Shatapushpa Choorna | Hingutriguna taila |
| Mahanarayana taila | Dhanwantaram chikkanapaka |
| Punarnavasavam | Satapushpa taila |
| Sathavaree ghritam | Kallyanagulam |
| Sukumara rasayana | Monthwise different ksheera pakas |
| Mahakalyanaka ghrita | Dadimadi ghrita |
| Dhanwantara taila |  |
